# Supplementary material for: Splice-Junction-Based Mapping of Alternative Isoforms in the Human Proteome
Source: Cell Rep. Author manuscript; Available in PMC 2020 Jan 15. (PMC6961840; doi:10.1016/j.celrep.2019.11.026)
Supplement: 2 [file NIHMS1546469-supplement-2.zip › DF1/PXD010154/Testis/Testis_20_MYO6_NDGTRPKMTPHPTDGFQMGNIVDIGPDSLTIEPLNQK.pdf]

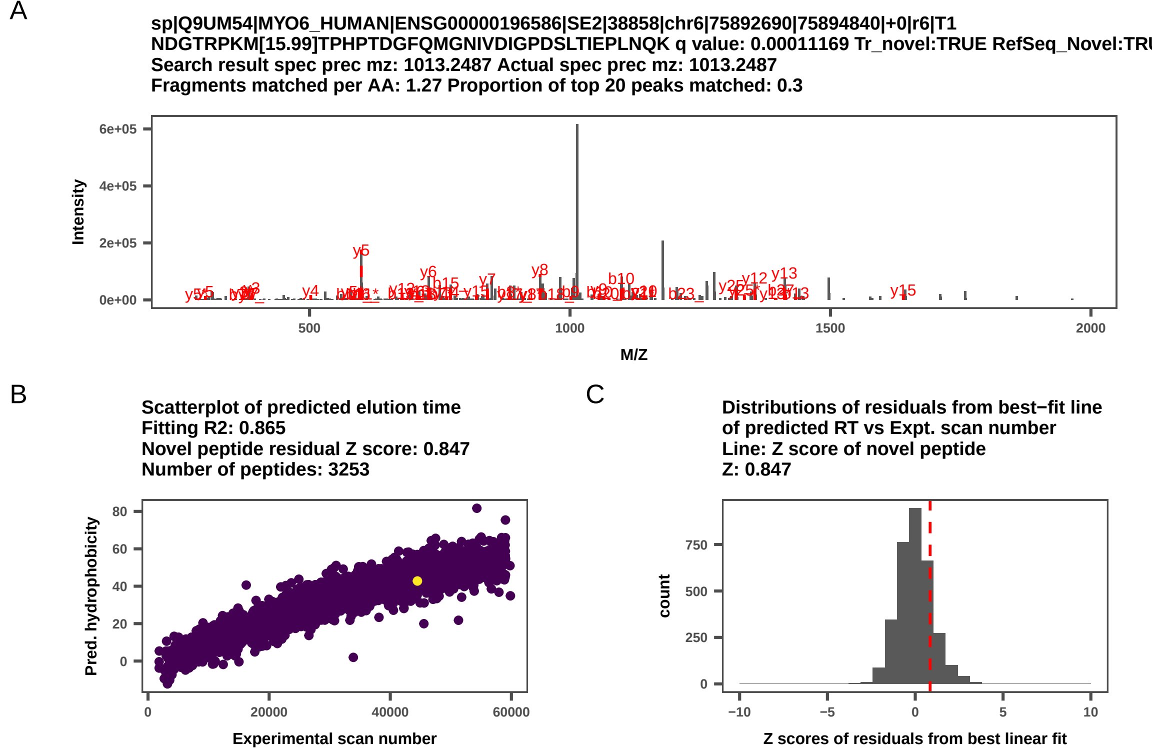
A
sp|Q9UM54|MYO6_HUMAN|ENSG00000196586|SE2|38858|chr6|75892690|75894840|+0|r6|T1
NDGTRPKM[15.99]TPHPTDGFQMGNIVDIGPDSLTIEPLNQK q value: 0.00011169 Tr_novel:TRUE RefSeq_Novel:TRU
Search result spec prec mz: 1013.2487 Actual spec prec mz: 1013.2487
Fragments matched per AA: 1.27 Proportion of top 20 peaks matched: 0.3
Intensity
6e+05
4e+05
2e+05
0e+00
500
1000
1500
2000
M/Z
B
Scatterplot of predicted elution time
Fitting R2: 0.865
Novel peptide residual Z score: 0.847
Number of peptides: 3253
Pred. hydrophobicity
80
60
40
20
0
0
20000
40000
60000
Experimental scan number
C
Distributions of residuals from best−fit line
of predicted RT vs Expt. scan number
Line: Z score of novel peptide
Z: 0.847
count
750
500
250
0
−10
−5
0
5
10
Z scores of residuals from best linear fit
